# Atopic Diseases and Systemic Lupus Erythematosus: An Epidemiological Study of the Risks and Correlations

**DOI:** 10.3390/ijerph110808112

**Published:** 2014-08-08

**Authors:** Yu-Ping Hsiao, Jeng-Dau Tsai, Chih-Hsin Muo, Chung-Hung Tsai, Fung-Chang Sung, Ya-Tang Liao, Yen-Jung Chang, Jen-Hung Yang

**Affiliations:** 1 Institute of Medicine, School of Medicine, Chung Shan Medical University, Taichung 402, Taiwan; E-Mails: missyuping@gmail.com (Y.-P.H.); fernand.tsai@msa.hinet.net (J.-D.T.); patholog@csmu.edu.tw (C.-H.T.); 2 Department of Dermatology, Chung Shan Medical University Hospital, Taichung 402, Taiwan; 3 Department of Pediatrics, Chung Shan Medical University Hospital, Taichung 402, Taiwan; 4 Institute of Clinical Medical Science, China Medical University College of Medicine, Taichung 404, Taiwan; E-Mails: a17776@mail.cmuh.org.tw (C.-H.M.); fcsung1008@yahoo.com (F.-C.S.); 5 Management Office for Health Data, China Medical University Hospital, Taichung 404, Taiwan; E-Mail: yatang.liao@gmail.com; 6 Department of Health Promotion and Health Education, National Taiwan Normal University, Taipei 106, Taiwan; 7 School of Medicine, Tzu Chi University, and Department of Dermatology, Buddhist Tzu Chi General Hospital, Hualien 907, Taiwan

**Keywords:** systemic lupus erythematosus, atopic disease, case-control study, insurance data, Taiwan

## Abstract

Both atopic diseases and systemic lupus erythematosus (SLE) are immune disorders that may lead to physical complications or multi-system comorbidities. This population-based case-control study was designed to evaluate the risk of SLE associated with atopic diseases. Using a national insurance claims dataset in Taiwan, we identified 1673 patients newly diagnosed with SLE and 6692 randomly selected controls frequency matched for gender, age and index date. The odds ratios (OR) for SLE were calculated for associations with allergic rhinitis, allergic conjunctivitis, atopic dermatitis and asthma. The SLE patients were predominantly female (82.5%) with a mean age of 40.1 (SD = 18.2). The patients with SLE had a higher rate of atopic dermatitis (6.81% *vs.* 3.06%), and asthma (10.6% *vs.* 7.64%) was approximately 2 times more common in the patients with lupus than in those without. The patients with atopic disease (atopic dermatitis, allergic rhinitis, allergic conjunctivitis and asthma) were at a significant risk for SLE. The overall risk for SLE increased as the number of atopic diseases increased from 1.46 to 2.29, compared with—individuals without the diseases (*p* < 0.0001). In conclusion, this population-based case-control study demonstrates a significant relationship between atopic diseases and the risk of SLE, especially for females. Atopic dermatitis plays a stronger role than other types of atopic disease in association with SLE.

## 1. Introduction

Atopic diseases (AD) are common chronic conditions in the general population. They are provoked by allergens and usually manifest as recurring, non-infectious, inflammatory conditions that begin in childhood. A number of genetic and environmental trigger factors are known to be involved in systemic lupus erythematosus (SLE). Some studies have focused on the associations of SLE with genes, but not all patients with the pathological alleles have SLE [1,2,3]. AD, such as atopic dermatitis, allergic conjunctivitis, allergic rhinitis and asthma, are associated with a hyperreactivity towards environmental antigens and food allergens. The associations between SLE and AD have previously been shown to be statistically correlated [3,4,5].

Both AD and SLE are immune diseases involving gene-environment interactions [1,4]. Previous research has shown epidemiological correlations and a substantial pathophysiological relationship between AD and SLE [3,4,5]. In patients with AD, variable autoantibody and antinuclear antibodies (ANA) have been identified [6,7]. The correlations between AD and SLE have been investigated from bench to bedside to clarify the immune-gene interactions, even though very few studies have demonstrated the epidemiological association between AD and SLE [8]. Using insurance data we conducted a case-control study to investigate the risk of SLE associated with AD, including allergic rhinitis, allergic conjunctivitis, atopic dermatitis and asthma.

## 2. Material and Methods

### 2.1. Data Source

We obtained the Longitudinal Health Insurance Database 2000 (LHID 2000) from the National Health Research Institutes. This database includes the scrambled identification codes, personal information and medical records from 1996 to 2010 of one million beneficiaries who were randomly selected from the original registry of beneficiaries in the Taiwan Health Insurance program. The age and gender distributions were not different between the LHID 2000 and the registry of beneficiaries. The registry of beneficiaries covered 96.6% of Taiwan’s population in 2000, and there are currently more than 23 million people enrolled in the program, which represented 99.6% of the Taiwanese population in 2011 [9]. The International Classification of Diseases, 9th Revision, Clinical Modification (ICD-9-CM) was used to identify the diagnoses of diseases in the claims data due to government policy. For the protection of privacy, the identities of the patients, physicians and institutions were scrambled in accordance with the Personal Electronic Data Protection Law. All subjects gave their informed consent for inclusion before they participated in the study. The study was conducted in accordance with the Department of Public Health, China Medical University, and Management Office for Health Data, China Medical University Hospital, and the protocol was approved by the institutional review board at the China Medical University Hospital (Project identification code: IRB-CMU-REC-101-012)”.

### 2.2. SLE cases and Their Controls

**Figure 1:**
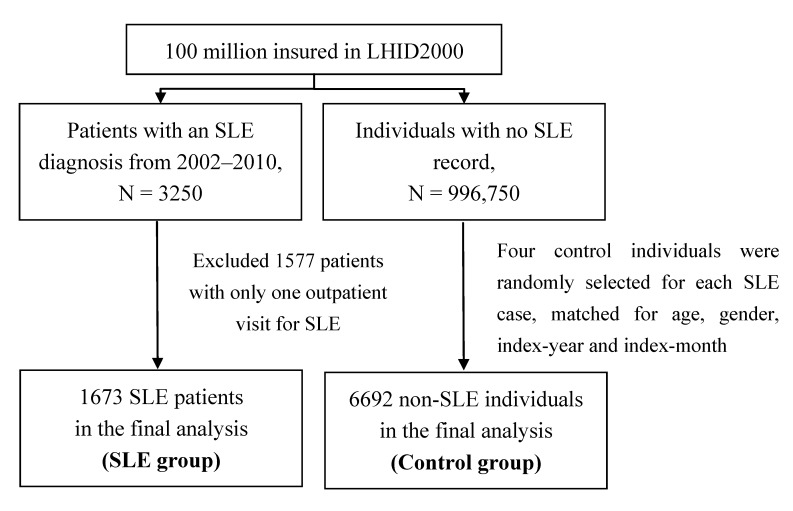
Flow chart for the selection of study subjects.

In this study, we identified 3250 patients newly diagnosed with SLE in 2002–2010 from the Taiwanese national health insurance claims data from the LHID 2000 (ICD-9-CM code: 373.34, 695.4 and 710.0). The Taiwan Rheumatology association [10] follows the “Eleven Criteria of Lupus” based on the American College of Rheumatology, and the assessments of SLE and AD were made by the rheumatologists and physicians in charge at their respective medical institutions. The Eleven Criteria of Lupus include malar rash, discoid rash, photosensitivity, mouth or nose ulcers, arthritis, pericarditis or lungs, neurologic disorder, nephritis, hematologic disorder, immunologic disorder, and antinuclear antibodies (ANA). Other complicated connective tissue diseases such as dermatomyositis, Sjogren’s syndrome, and scleroderma were excluded in this study. All the diseases were defined by at least one inpatient visit or two outpatient visits. After excluding cases with only one record, 1673 subjects newly diagnosed with SLE were defined as cases with the date of diagnosis of SLE as the index date. In order to have a greater power of statistical tests, four control subjects were randomly selected for each SLE case, frequency matched by age (every 5 years), gender, index-year and index-month (6692 controls in total). None of the controls had a history of SLE (Figure 1). The information on the history of atopic diseases and the degree of urbanization where the study subjects resided was also extracted from claims data for this study.

### 2.3. Atopic Diseases and Covariates

The atopic diseases, including allergic rhinitis (ICD-9-CM code: 477), atopic conjunctivitis (ICD-9-CM code: 372.05, 372.10 and 372.14), atopic dermatitis (ICD-9-CM code: 691.8) and asthma (ICD-9-CM code: 493), were retrospectively identified by at least one inpatient visit or two outpatient visits with the diagnosis appearing in either the primary or secondary coding fields. The age, gender and urbanization levels (level 1 was the highest level of urbanization and level 5 was the lowest) were also included in the variables of interest.

### 2.4. Statistical Analysis

Distributions of demographic status and AD were compared between cases and controls examined using Chi-square test for the categorical variables and t-test for the continuous variables. The odds ratios (ORs) and 95% confidence intervals (CIs) were also estimated using unconditional logistic regression analysis that took into account the frequency matched case-control design and the multivariate model. The stratified analyses were further performed to compared age adjusted ORs of lupus by AD type and number of AD between males and females. Data analysis also plotted the proportional distribution of SLE and AD by age, and ORs of SLE by age and number of ADs. The analyses were performed using SAS software, version 9.2 (SAS Institute Inc., Cary, NC, USA). A *p*-value of 0.05 was considered significant.

## 3. Result

The demographic characteristics of the patients with SLE and the non-SLE controls are shown in Table 1. There were 1673 SLE patients and 6692 non-SLE controls included in this study. There were more females than males (82.5% *vs.* 17.5%), and the 20 to 30-year-old age range had the highest proportion of SLE cases (mean age = 40.1, SD = 18.2). The SLE patients had more atopic disease comorbidities than the controls. The atopic diseases, including allergic rhinitis (OR = 1.52, 95% CI: 1.34–1.73), allergic conjunctivitis (OR = 1.53, 95% CI: 1.37–1.72), atopic dermatitis (OR = 2.31, 95% CI: 1.83–2.93) and asthma (OR = 1.43, 95% CI: 1.20–1.71) were associated with an increased risk of SLE.

**Table 1:** Comparison in demographic characteristics and atopic diseases between SLE cases and controls.

Demographic Characteristics	Controls N = 6692		Cases N = 1673		cOR	95% CI
	N	%	N	%		
**Sex**						
Female	5524	82.5	1381	82.5	1.00	(Reference)
Male	1168	17.5	292	17.5	1.00	(0.87–1.15)
**Age, years**						
<20	964	14.4	241	14.4	1.00	(Reference)
20–39	2596	38.8	649	38.8	1.00	(0.85–1.18)
40–59	2108	31.5	527	31.5	1.00	(0.84–1.19)
≥60	1024	15.3	256	15.3	1.00	(0.82–1.22)
Mean (SD)	40.0	(18.4)	40.1	(18.2)		
**Urbanization ^†^**						
1	2004	30.0	532	31.8	1.17	(0.99–1.37)
2	1985	29.7	487	29.1	1.08	(0.92–1.27)
3	1206	18.0	274	16.4	1.00	(reference)
4	910	13.6	223	13.3	1.08	(0.89–1.31)
5	587	8.77	157	9.38	1.18	(0.95–1.47)
**Comorbidity ^†^**						
Allergic rhinitis	1274	19.0	441	26.4	1.52	(1.34–1.73) ***
Allergic conjunctivitis	1613	24.1	548	32.8	1.53	(1.37–1.72) ***
Atopic dermatitis	205	3.06	114	6.81	2.31	(1.83–2.93) ***
Asthma	511	7.64	177	10.6	1.43	(1.20–1.71) ***	

Note: **^†^** Chi-square test *p* < 0.0001; *** *p* < 0.001.

Table 2 presents the association between SLE and the atopic diseases compared between males and females. Overall, the subjects with atopic dermatitis (OR = 2.13, 95% CI: 1.67–2.70) had the highest risk of SLE, followed by allergic conjunctivitis (OR = 1.43, 95% CI: 1.26–1.61) and allergic rhinitis (OR = 1.36, 95% CI: 1.19–1.55). These same results were observed for the female subjects. However, atopic dermatitis was only associated with an increased risk of SLE (OR = 2.00, 95% CI: 1.01–3.99) with marginal statistical significance in the male subjects. The overall risk for SLE increased with the number of atopic diseases, increasing from 1.46 to 2.29 when compared with study subjects without any comorbidities (*p* < 0.0001). Both genders showed the same trends.

**Table 2:** Logistic regression estimated odds ratio of SLE associated with atopic diseases by gender.

Atopic Diseases	All		Female		Male	
	OR	(95% CI)	OR	(95% CI)	OR	(95% CI)
**Atopic diseases ^†^**						
Allergic rhinitis	1.36	(1.19–1.55) ***	1.39	(1.20–1.60) ***	1.22	(0.88–1.68)
Allergic conjunctivitis	1.43	(1.26–1.61) ***	1.44	(1.27–1.64) ***	1.32	(0.95–1.83)
Atopic dermatitis	2.13	(1.67–2.70) ***	2.14	(1.66–2.76) ***	2.00	(1.01–3.99) *
Asthma	1.18	(0.97–1.43)	1.21	(0.97–1.49)	1.04	(0.66–1.64)
**Number of atopic diseases**						
0	1.00	(reference)	1.00	(reference)	1.00	(reference)
1	1.46	(1.29–1.65) ***	1.51	(1.32–1.73) ***	1.24	(0.92–1.67)
2	2.11	(1.79–2.49) ***	2.24	(1.87–2.68) ***	1.54	(1.04–2.37) *
≥3	2.29	(1.71–3.06) ***	2.33	(1.71–3.19) ***	2.13	(0.95–4.78)
*p* for trend	<0.0001	<0.0001	0.007			

Notes: **^†^** Adjusted for age and four mutual atopic diseases; * *p* < 0.05, *** *p* < 0.001.

**Figure 2:**
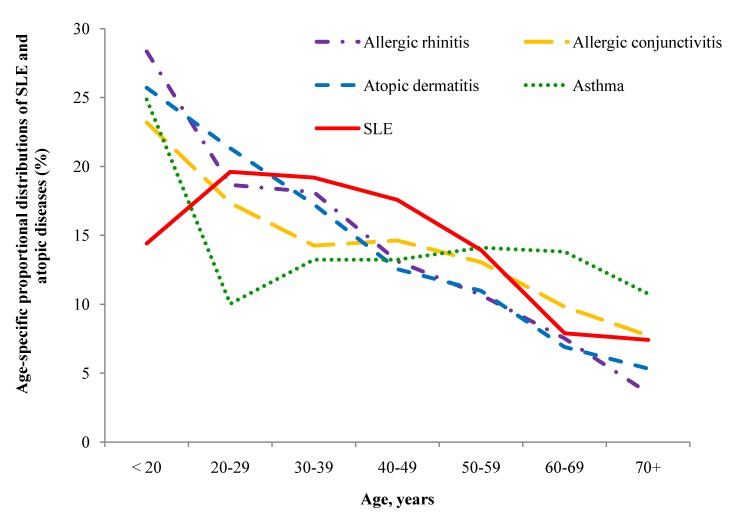
Age-specific proportional distributions of SLE and atopic diseases, including atopic dermatitis, allergic rhinitis, allergic conjunctivitis, atopic dermatitis and asthma.

**Figure 3:**
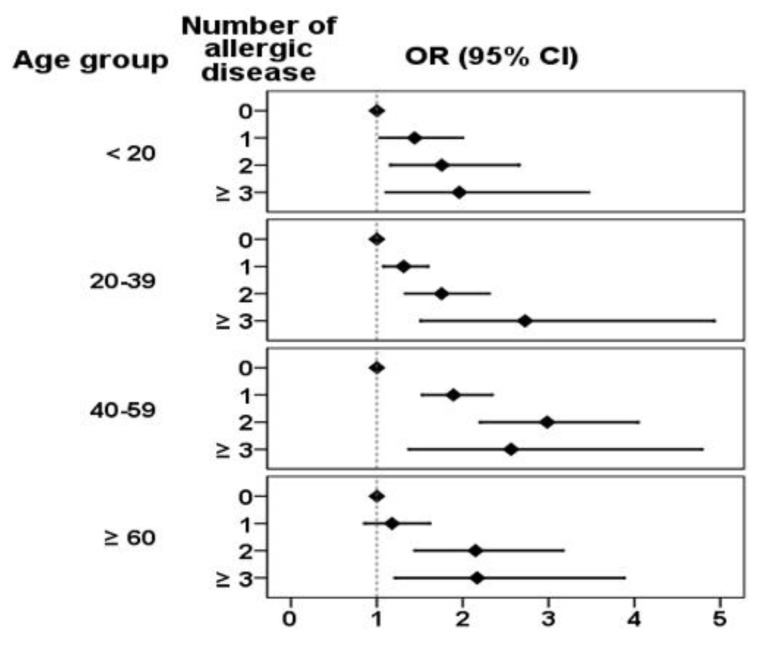
Logistic regression estimayed odds ratio of SLE by age and number of atopic disease.

The proportion of individuals with SLE and atopic disease as they are distributed by age is shown in Figure 2. The highest portion of SLE appeared in the those aged 20–39 years. In general, specific AD decreased with age, although a plateau existed for asthma in those aged 30–69 years. Figure 3 shows the adjusted risk for SLE with increased numbers of atopic diseases in the different age groups. Compared with the controls, the risks for SLE were all significantly increased with numbers of atopic diseases in the below 60 age groups.

## 4. Discussion

The strength of this study lies in the nationwide population-based case-control database. The high recruitment rate of the nationwide database and the case control design, suggesting a high validity and extended availability, enabled us to eliminate possible coding biases from the insurance system. The current results demonstrate strong and independent associations between SLE and atopic diseases, adding substantial evidence of the reported relationship. The correlations were independent of various factors that are known to have an impact on the presence of SLE; here, we also demonstrated the associations correlated with the numbers of atopic diseases even though the severity of the disorders was not available from the LHID database.

The etiologies of both the atopic diseases and SLE are multi-factorial and heterogeneous, which contributes to their development. Epidemiological studies and case series reports have shown significant associations between allergic diseases and SLE [3,5,11,12,13]. In contrast, some studies on the relationship between SLE and AD have been controversial [14]. However, their case numbers were small, and the use of retrospective questionnaire studies is susceptible to recall bias, selection bias and exposure misclassification. The current study demonstrates that SLE was significantly associated with atopic dermatitis, allergic conjunctivitis, allergic rhinitis, and asthma. The risk of SLE was strongly correlated with the numbers of AD, indicating a strong association between the two immunologic diseases.

SLE and atopy share several pathophysiological mechanisms. In 1976, Goldman *et al.* first described that patients with SLE (N = 27) were prone to have allergic rhinitis and drug allergies than non-SLE controls [12]. Sequeira *et al.* found that drug, skin and insect allergies were particularly frequent in patients with SLE (N = 132), and 63% of the SLE patients had at least one type of allergic disorder [3,14]. The dysregulation of the immune system with the activation of B-cells leading to the production of immunoglobulins and autoantibodies plays an important role in SLE and atopic diseases [3]. The pathogenic autoantibodies arise by complex immune mechanisms, resulting in inflammation and tissue damage [1,3,4]. Several autoantibodies, such as antinuclear antibodies, anti-SSA, anti-RNP, anti-elongation factor-1a, anti-DFS 70 kd/transcription coactivator p75 have been detected in serum of AD patients [5,15,16].

The plausible postulated mechanism was that experimentally elicited IgE autoantibodies and/or IgG autoantibodies provoked eczematous lesions in atopic dermatitis [11]. Approximately 25% of severe AD patients had IgE autoantibodies against proteins from keratinocytes and endothelial cells [17]. Based on the definition of “autoreactivity” as *in vitro* or *in vivo* evidence of immune response to autologous, generic, or recombinant human proteins or other tissue/cellular components, the prevalence of “autoreactivity” ranged from 23% to 91% in AD patients in 14 studies involving 2644 participants. Therefore, the hypothesis that ‘‘autoreactivity’’ plays a role in AD is based on two major observations. First, the chronic relapsing-remitting pattern of AD is similar to that of other autoimmune diseases. Second, autologous or human components elicit immediate hypersensitive reactions in AD [11]. Approximately 20% to 30% of AD patients have ANA, ranging in titer between 1:40 and 1:1280 [5]. It was suggested that the ANA (+) AD patients should be carefully followed up in the long-term to monitor the development of SLE manifestations if other autoantibodies are positive [5]. However, the role of autoantibodies contributing to atopic diseases requires further investigation [11].

The contribution of IgE to lupus pathogenesis remains uncertain. IgE may play a role in the active phase of SLE [3], but Parks *et al.* assessed total serum IgE levels and atopy in SLE patients and found no evidence of an independent association between the IgE levels and SLE [18]. The immunological hallmark of IgE-mediated allergy is IgE sensitization, which in the vast majority of epidemiological studies has been found to be more common in males than in females [19]. That does not really fit with the hypothesis that atopic disease increases the risk of autoimmunity and SLE, because SLE is far more common in females. However, this does not in any way rule out that atopic disease could play a role in SLE risk. Feizy *et al.* observed that atopic diseases are not uncommon in a proportion of autoimmune diseases, e.g., SLE with Th2 overactivity [19]. By a molecular survey, an overlap of autoimmune/inflammatory loci on chromosome 1p22.3–p22.1 and 19p13 was identified in both AD and SLE. It was postulated that genetic components may not be tissue specific but may share basic mechanisms of immune regulation [20].

The average annual incidence of SLE was 8.1 per 100,000 in Taiwan and the prevalence increased steadily from 42.2 cases per 100,000 persons in 2000 to 67.4 cases per 100,000 persons in 2007 in a recent study [21]. With regard to the age distribution of SLE and AD, they showed different incidence distribution patterns [6,7]. In the current study, the ages specific distributions of ADs showed that Children accounted the highest portion and then declined rapidly by age. However, the ages specific distributions is somewhat of difference from other types of AD. The asthmatic cases in children are at the peak among age groups, which drops sharply to deep in young adults. In contrast, the incidence of SLE is relatively low in children but increases to a peak in those 20 to 40 years of age. This may indicate that the youth are at higher risk of ADs to develop SLE in adulthood. This may match a developmental pattern for a cluster of ADs that begin in the early childhood. SLE thus becomes common in the early adulthood and declines as all ADs decline with age.

The gender ratio of SLE in Taiwan was similar to that of Caucasians, with a marked female predominance, especially in patients 20 to 54 years old [21,22]. The present study showed no statistical significance in SLE risk among urbanization levels. Females with atopic dermatitis had a higher association with SLE (OR = 2.14, 95% CI = 1.66–2.76), followed by allergic conjunctivitis and allergic rhinitis. However, in males, only those patients with atopic dermatitis had a marginally increased risk for SLE (OR = 2.00, 95% CI = 1.01–3.99) (Table 2). Estrogen may regulate the expression of and responsiveness to autoantigens in SLE and AD; this may be associated with the hyperreactivity to exogenous antigens [23]. Therefore, we suggest that the correlation between AD and SLE is more prominent in the female population. In addition, the overall risk for SLE was increased with the number of atopic diseases in both genders and in the different age groups. The underlying pathophysiology responsible for the higher association with more atopic diseases and SLE needs further study.

Limitations of this study include that the registry-based data may underestimate the prevalence of patients and that the information on the clinical conditions including the laboratory data, and the severity of SLE are not available. Patients with SLE included in our study were given a Catastrophic Illness code by board-certified physicians in their corresponding specialties; Berkson’s bias and the misclassification of the illness may exist. It is also possible that atopic diseases may have been falsely diagnosed in individuals with SLE. This may be supported by the finding that atopic dermatitis is the disease showing the strongest association with SLE, because SLE can also manifest as a skin rash. Other associated connective tissue diseases were not excluded from this study, nor were they able to differentiate pre-existing SLE or the incidence of SLE after AD. These results do not clarify whether treatment should take place during a subclinical state or latent period or whether this would influence the results.

In conclusion, this epidemiologic study has demonstrated significant associations between AD and the risk of SLE, implying a shared autoimmune mechanism and evolutionary condition.
